# Wing morphometrics as a tool in species identification of forensically important blow flies of Thailand

**DOI:** 10.1186/s13071-017-2163-z

**Published:** 2017-05-10

**Authors:** Narin Sontigun, Kabkaew L. Sukontason, Barbara K. Zajac, Richard Zehner, Kom Sukontason, Anchalee Wannasan, Jens Amendt

**Affiliations:** 1 Department of Parasitology, Faculty of Medicine, Chiang Mai University, Chiang Mai, 50200 Thailand; 2 Institute of Legal Medicine, Forensic Biology/Entomology, Kennedyallee 104, 60596 Frankfurt am Main, Germany

**Keywords:** Species identification, Forensic entomology, Wing morphometry, Blow fly, Thailand

## Abstract

**Background:**

Correct species identification of blow flies is a crucial step for understanding their biology, which can be used not only for designing fly control programs, but also to determine the minimum time since death. Identification techniques are usually based on morphological and molecular characters. However, the use of classical morphology requires experienced entomologists for correct identification; while molecular techniques rely on a sound laboratory expertise and remain ambiguous for certain taxa. Landmark-based geometric morphometric analysis of insect wings has been extensively applied in species identification. However, few wing morphometric analyses of blow fly species have been published.

**Methods:**

We applied a landmark-based geometric morphometric analysis of wings for species identification of 12 medically and forensically important blow fly species of Thailand. Nineteen landmarks of each right wing of 372 specimens were digitised. Variation in wing size and wing shape was analysed and evaluated for allometric effects. The latter confirmed the influence of size on the shape differences between species and sexes. Wing shape variation among genera and species were analysed using canonical variates analysis followed by a cross-validation test.

**Results:**

Wing size was not suitable for species discrimination, whereas wing shape can be a useful tool to separate taxa on both, genus and species level depending on the analysed taxa. It appeared to be highly reliable, especially for classifying *Chrysomya* species, but less robust for a species discrimination in the genera *Lucilia* and *Hemipyrellia.* Allometry did not affect species separation but had an impact on sexual shape dimorphism.

**Conclusions:**

A landmark-based geometric morphometric analysis of wings is a useful additional method for species discrimination. It is a simple, reliable and inexpensive method, but it can be time-consuming locating the landmarks for a large scale study and requires non-damaged wings for analysis.

**Electronic supplementary material:**

The online version of this article (doi:10.1186/s13071-017-2163-z) contains supplementary material, which is available to authorized users.

## Background

Blow flies are considered to be of medical importance worldwide. Adults are mechanical vectors of several pathogens in humans, i.e. viruses, bacteria, protozoan cyst, helminth eggs, and fungi [1–4]. Their larvae are myiasis-producing agents in living humans and vertebrate animals, particularly the genera *Cochliomyia*, *Chrysomya*, *Lucilia* and *Calliphora* [5–7]. In addition, blow flies are forensically important insects as immature stages feed on human corpses and can be used in forensic investigations [8–10]. Forensic entomology is the analysis of insect evidence for forensic and legal purposes and is most frequently used for the estimation of the minimum time since death (PMI_min_) [11].

Correct species identification of the blow fly is a crucial step in understanding its biology for not only designing fly control programs but also determining the PMI_min_ precisely. Misidentification may impact the effectiveness of fly control strategies and bias the calculation of developmental times, eventually leading to an incorrect PMI_min_. Several identification techniques based on morphological [12, 13] and molecular characters exist [14, 15]. However, the use of classical morphology such as bristles on the body or male genitalia are very difficult to apply for non-experts and DNA identification can still remain ambiguous as, for example, some forensically important fly species are not, or insufficiently, represented in the reference libraries [16], many of the existing sequences in those online libraries just represent “dark taxa”, i.e. they are not identified to species level [17], and DNA barcodes failed to distinguish among certain closely related species [14, 18–21]. This problem is even more serious in regions, where the accurate knowledge of relevant species is a challenge.

Besides classical morphology or DNA identification, the use of morphometrics has shown to be a valuable tool for interspecific discrimination. Morphometrics is defined as the quantitative studies of biological size and shape, shape variation, and its covariation with other biotic or abiotic factors [22] and can be a valuable tool for interspecific discrimination. In recent years, a landmark-based geometric morphometric analysis of insect wings has been extensively applied in entomology, particularly in taxonomy [23–26] and geographic variation of species [27–29] due to its simplicity, low costs and high reliability. Several orders have been studied such as the Diptera [23, 30], Hymenoptera [31], Coleoptera [26] and Odonata [32].

However, just a few wing morphometric analyses of blow fly species have been published [24, 28, 33], and the focus of such analysis for identification was mainly on medical and not in forensic entomology [28, 34, 35]. Therefore, the aim of the study is to test a landmark-based geometric morphometric analysis of wings for species identification of medically and forensically important blow flies of Thailand, one of the global hotspots of biodiversity.

## Methods

### Specimen collection

**Fig. 1:**
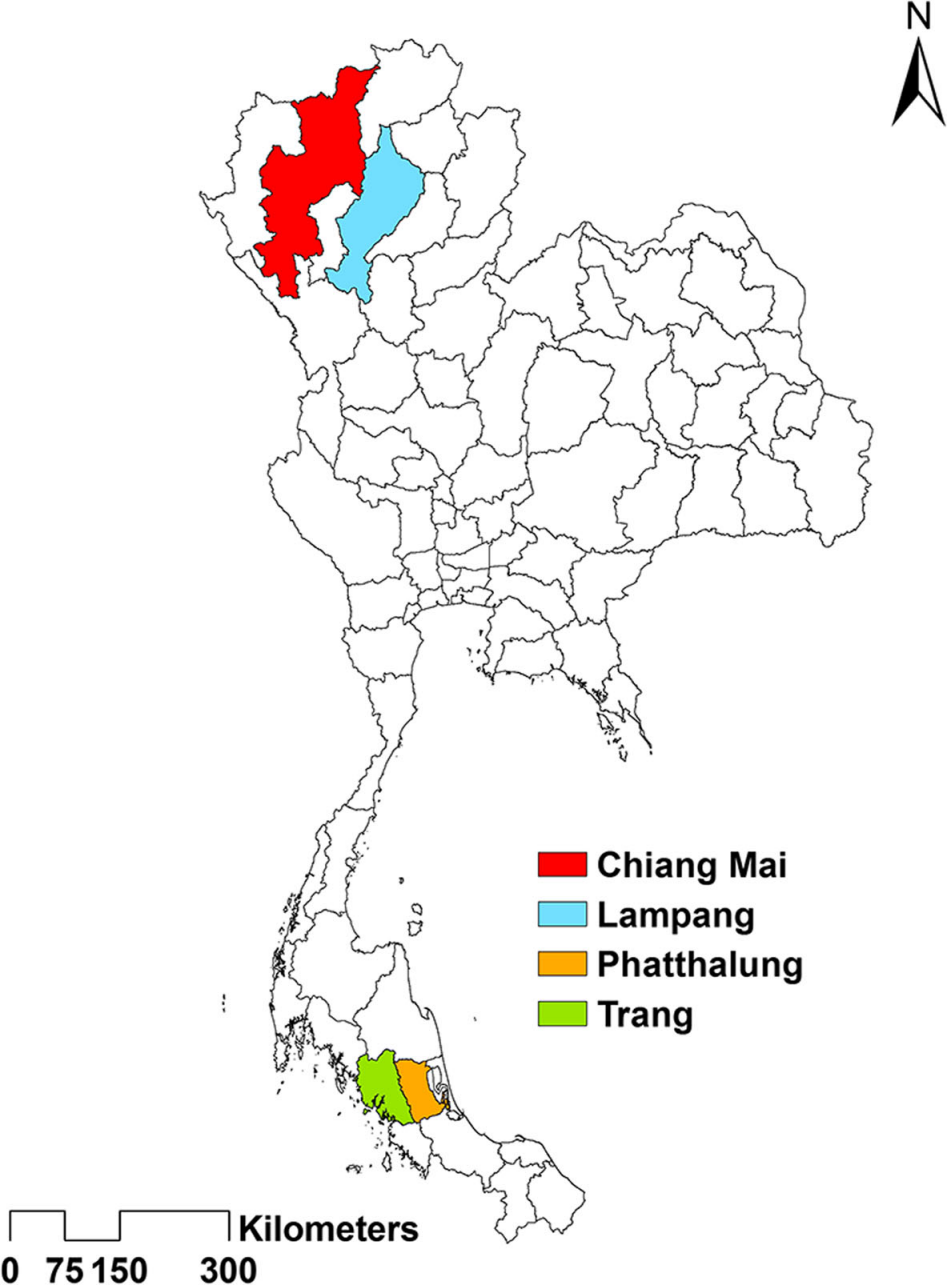
Map of Thailand showing provinces of collection sites for adult blow flies used for wing morphometric analysis

Adult blow flies used in this study were collected during 2013–2014 in several locations of Thailand (Table 1, Fig. 1). A total of 372 blow flies were captured by using semi-automatic funnel trap invented by Kom Sukontason (Additional file 1), or by using a hand-held fly net, sweeping it over a bait of 1-day-old beef offal (~300 g). Specimens were either killed by ethyl acetate in the field or kept alive in the same fly net used to catch the flies and then transported back to the laboratory within 1 h and frozen at -20 °C for 2 h. Specimens were pinned and identified based on the taxonomic key of Kurahashi and Bunchu [13]. Sampled specimens belong to 12 species including *Chrysomya megacephala*, *Chrysomya chani*, *Chrysomya pinguis*, *Chrysomya rufifacies*, *Chrysomya villeneuvi*, *Chrysomya nigripes*, *Lucilia cuprina*, *Lucilia papuensis*, *Lucilia porphyrina*, *Lucilia sinensis*, *Hemipyrellia ligurriens* and *Hemipyrellia pulchra* (Table 1), therefore including the most relevant taxa from medical and forensic point of views.

**Table 1:** List of Thai blow fly specimens used in this study

Subfamily	Species	Code of specimens	Province (Location)	GPS reference		Total no. of specimens (males/females)
				Latitude	Longitude	
Chrysomyinae	*Chrysomya megacephala*	CM	Chiang Mai (Ban Pang Daeng)	19°0′2.986″N	99°17′16.016″E	4 (4/0)
			Chiang Mai (Ban Pang Mai Daeng)	19°8′27.051″N	98°52′16.893″E	4 (4/0)
			Chiang Mai (Doi Nang Kaew)	19°3′52.991″N	99°22′34.015″E	5 (1/4)
			Laboratory colony (origin Chiang Mai)	–	–	18 (9/9)
			Lampang (Doi Khun Tan)	18°23′34.837″N	99°12′54.186″E	5 (3/2)
			Phatthalung (Khao Aok Talu)	7°37′31.189″N	100°5′28.266″E	6 (1/5)
			Phatthalung (Khaojeak)	7°36′37.134″N	100°1′58.433″E	9 (1/8)
			Trang (Yantakhao)	7°24′1.032″N	99°40′28.441″E	2 (1/1)
		Total				53 (24/29)
	*Chrysomya chani*	CC	Chiang Mai (Ban Pang Daeng)	19°0′2.986″N	99°17′16.016″E	15 (6/9)
			Chiang Mai (Ban Pang Mai Daeng)	19°8′27.051″N	98°52′16.893″E	7 (4/3)
			Chiang Mai (Doi Nang Kaew)	19°3′52.991″N	99°22′34.015″E	4 (4/0)
			Chiang Mai (forest area, Mae Hia)	18°46′01.08″N	98°56′08.3″E	9 (0/9)
			Lampang (Doi Khun Tan)	18°23′34.837″N	99°12′54.186″E	5 (3/2)
		Total				40 (17/23)
	*Chrysomya pinguis*	CP	Chiang Mai (Doi Nang Kaew)	19°3′52.991″N	99°22′34.015″E	30 (20/10)
			Lampang (Doi Khun Tan)	18°23′34.837″N	99°12′54.186″E	9 (9/0)
		Total				39 (29/10)
	*Chrysomya rufifacies*	CR	Chiang Mai (Ban Pang Daeng)	19°0′2.986″N	99°17′16.016″E	30 (15/15)
			Chiang Mai (Ban Pang Mai Daeng)	19°8′27.051″N	98°52′ 16.893″E	9 (9/0)
			Chiang Mai (Doi Nang Kaew)	19°3′52.991″N	99°22′ 34.015″E	8 (1/7)
		Total				47 (25/22)
	*Chrysomya villeneuvi*	CV	Chiang Mai (Doi Nang Kaew)	19°3′52.991″N	99°22′34.015″E	8 (5/3)
			Chiang Mai (forest area, Mae Hia)	18°46′01.08″N	98°56′08.3″E	22 (9/13)
			Lampang (Doi Khun Tan)	18°23′ 34.837″N	99°12′ 54.186″E	9 (9/0)
		Total				39 (23/16)
	*Chrysomya nigripes*	CN	Chiang Mai (forest area, Mae Hia)	18°46′01.08″N	98°56′08.3″E	32 (17/15)
		Total				32 (17/15)
Luciliinae	*Lucilia cuprina*	LC	Laboratory colony (origin Chiang Mai)	–	–	29 (15/14)
		Total				29 (15/14)
	*Lucilia papuensis*	LPA	Chiang Mai (Doi Nang Kaew)	19°3′52.991″N	99°22′34.015″E	9 (2/7)
			Chiang Mai (forest area, Mae Hia)	18°46′01.08″N	98°56′08.3″E	21 (5/16)
			Lampang (Doi Khun Tan)	18°23′34.837″N	99°12′54.186″E	2 (2/0)
		Total				32 (9/23)
	*Lucilia porphyrina*	LPO	Chiang Mai (Doi Nang Kaew)	19°3′52.991″N	99°22′34.015″E	10 (8/2)
			Chiang Mai (San Ku, Doi Suthep-Pui Mountain)	18°48′56.307″N	98°53′ 40.782″E	8 (3/5)
		Total				18 (11/7)
	*Lucilia sinensis*	LS	Chiang Mai (forest area, Mae Hia)	18°46′01.08″N	98°56′08.3″E	3 (1/2)
			Chiang Mai (San Ku, Doi Suthep-Pui Mountain)	18°48′56.307″N	98°53′40.782″E	2 (2/0)
			Chiang Mai (Sirindhon Observatory)	18° 47′21.022″N	98°55′16.562″E	1 (1/0)
			Chiang Mai (Tham Phra Leu Sri)	18°48′20.252″N	98°54′34.238″E	2 (0/2)
		Total				8 (4/4)
	*Hemipyrellia ligurriens*	HL	Chiang Mai (forest area, Mae Hia)	18°46′01.08″N	98°56′08.3″E	32 (14/18)
		Total				32 (14/18)
	*Hemipyrellia pulchra*	HP	Chiang Mai (longan orchard, Mae Hia)	18°45′56.66″N	98°55′40.13″E	3 (0/3)
		Total				3 (0/3)

### Slide preparation

**Fig. 2:**
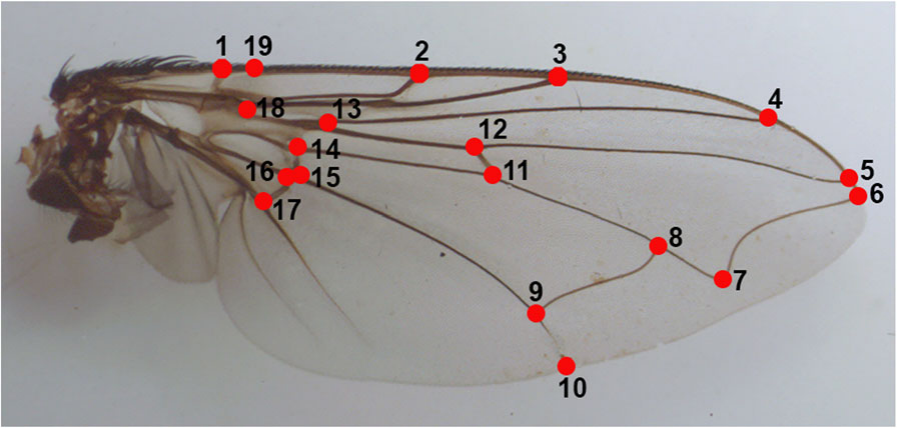
Right wing of female *Ch. chani* showing the 19 plotted landmarks based on Hall et al. [28]

The right wing of each fly was removed with fine forceps. A drop of Permount™ Mounting Medium was added on a microscope slide, and then the wing was placed onto the drop and covered with the cover slip. Before placing the wing onto the drop, the wing was submerged in xylene to facilitate the montage and avoid bubbles. All mounted wing slides were kept as thin as possible using the minimum mounting medium to maximise wing flattening and then dried at room temperature for a week. Each wing was photographed using a digital camera attached to a stereomicroscope at 1.5× magnification. Images were used to build tps files by using the TpsUtil V. 1.64 software [36] to minimise a possible bias when digitising the landmark locations. Altogether 19 landmarks as used by Hall et al. [28] (Fig. 2) were digitised using TpsDig2 V.2.20 software [37]. Each wing was digitised twice to reduce the measurement error [38].

### Geometric morphometric analysis

The tps files were subjected to the MorphoJ software [39], and then the raw landmark coordinates of all specimens were aligned and superimposed using Procrustes Fit function to remove variation due to differences in scale, position and orientation from the coordinates. The centroid size (the square root of the sum of the squared distances between the centre of the configuration of landmarks and each landmark) [40] and Procrustes coordinates obtained from landmark data were used for further statistical analyses. For establishing a possible measurement error, the Procrustes coordinates of each specimen were averaged after a generalised Procrustes analysis in MorphoJ. Centroid size was also averaged for each specimen.

### Size variation

Wing size was estimated by its centroid size [40]. Wing size difference among species was analysed using Kruskal-Wallis H-test, followed by Mann-Whitney *U*-test with Bonferroni correction applied for the significance level (0.05). Statistical analysis was performed in SPSS V.17.0 software for Windows (SPSS Inc., Chicago, Illinois, USA).

### Shape variation

Wing shape variation was analysed using MorphoJ software [39]. Canonical variate analysis (CVA) was used to determine the most important feature as a possible discriminator between groups (genera or species). The statistical significance of pairwise differences in mean shapes was analysed using permutation tests (10,000 rounds) with Mahalanobis distances and Procrustes distances. Additionally, a cross-validation test in discriminant function analysis (DFA) was used to assess the accuracy of classification based on Mahalanobis distances in a permutation test with 10,000 rounds using MorphoJ software [39].

### Sexual dimorphism

Sexual dimorphism consisted of sexual size dimorphism (SSD) and sexual shape dimorphism (SShD). Differences in size between sexes for each species were tested by Mann-Whitney *U*-test. Statistical analysis was performed in SPSS V.17.0 software for Windows (SPSS Inc., Chicago, Illinois, USA) at a significance level of 0.05. For wing shape dimorphism, DFA was performed, and shape differences between males and females of each species were estimated based on Mahalanobis distances in permutation test with 10,000 rounds using MorphoJ software [39]. In addition, cross-validation test was performed to assess the accuracy of the classification.

### Phenetic relationships of wing shape among blow fly species

To examine phenetic relationships among 12 blow fly species based on wing morphology, UPGMA (unweighted pair-group method with arithmetic averages) was performed using PAST V.3.09 software [41]. The UPGMA dendrogram was constructed based on Mahalanobis distances obtained by pairwise comparison of analysed species from CVA.

### Allometric effects

Allometry tries to describe how the characteristics of creatures change with size. For example, wing size sometimes affects wing shape variation (allometry) [34, 42]. To estimate such an allometric effect, the regression of Procrustes distance (dependent variable) on centroid size (independent variable) was analysed among species and within each species separately. Moreover, the sex-dependent effect of size on shape was analysed by a multivariate regression of shape, pooled within sex, on centroid size using MorphoJ software [39]. We also evaluated the effect of removing allometry on species and sex discrimination. The residuals from the regression of Procrustes coordinates on centroid size from the previous analyses were used for assessing the differences in shape without the size effect (allometry-free variables). The residuals from regression were subjected to a cross-validation test in DFA based on Mahalanobis distances in a permutation test with 10,000 rounds using MorphoJ software [39].

## Results

### Size variation

**Fig. 3:**
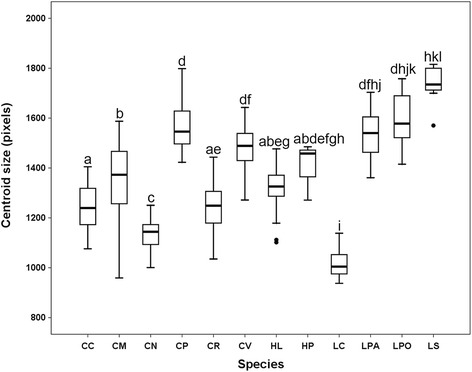
Boxplot showing centroid size of wings for each blow fly species; non-overlapping letters indicate a statistically significant difference (Mann-Whitney *U*-test, *P* < 0.0008). *Abbreviations*: CC, *Ch. chani*; CM, *Ch. megacephala*; CN, *Ch. nigripes*; CP, *Ch. pinguis*; CR, *Ch. rufifacies*; CV, *Ch. villeneuvi*; LC, *L. cuprina*; LPA, *L. papuensis*: LPO, *L. porphyrina*; LS, *L. sinensis*; HL, *He. ligurriens*; HP, *He. pulchra*. Each box shows the median as a vertical line across the middle, the quartiles (25th and 75th percentiles) at its ends, horizontal lines out the box indicate minimum and maximum, and outlier data are plotted as black circles

**Fig. 4:**
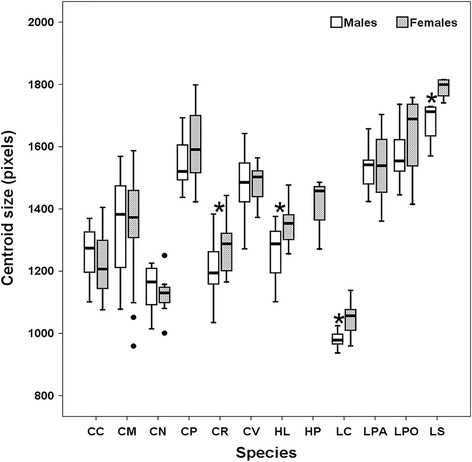
Boxplot showing centroid size of wings for each blow fly species (males and females). Only female specimens of *Hemipyrellia pulchra* were collected, thus it could not be used for classifying between sexes. Asterisks indicate statistically significant difference between males and females (Mann-Whitney *U*-test, *P* < 0.05). *Abbreviations*: CC, *Ch. chani*; CM, *Ch. megacephala*; CN, *Ch. nigripes*; CP, *Ch. pinguis*; CR, *Ch. rufifacies*; CV, *Ch. villeneuvi*; LC, *L. cuprina*; LPA, *L. papuensis*: LPO, *L. porphyrina*; LS, *L. sinensis*; HL, *He. ligurriens*. Each box shows the median as a vertical line across the middle, the quartiles (25th and 75th percentiles) at its ends, horizontal lines out the box indicate minimum and maximum, and outlier data are plotted as black circles

Centroid sizes among species were significantly different (Fig. 3, Kruskal-Wallis H-test: *χ* ^2^ = 286.222, *df* = 11, *P* = 0.000), but only the centroid sizes of *Ch. nigripes* and *L. cuprina* were significantly different from the other ten species (Mann-Whitney *U*-test, *P* < 0.0008). In this regard, the difference among species could not be explained by their wing size difference. For the effect of sexes on size, most species (7/11) showed no significant difference between males and females (Mann-Whitney *U*-test, *P* > 0.05), except for *Ch. rufifacies*, *L. cuprina*, *L. sinensis* and *He. ligurriens* (Mann-Whitney *U*-test, *P* < 0.05), which males were smaller than females (Fig. 4).

### Shape variation

The canonical variate analysis (CVA) was used to maximise variation between groups and minimise intraspecific variation. The shape difference in each genus or species on the shape space were scattered on the first two canonical variate axes (CV1 and CV2). The results from CVA were clearly discriminated in both genus and species.

**Fig. 5:**
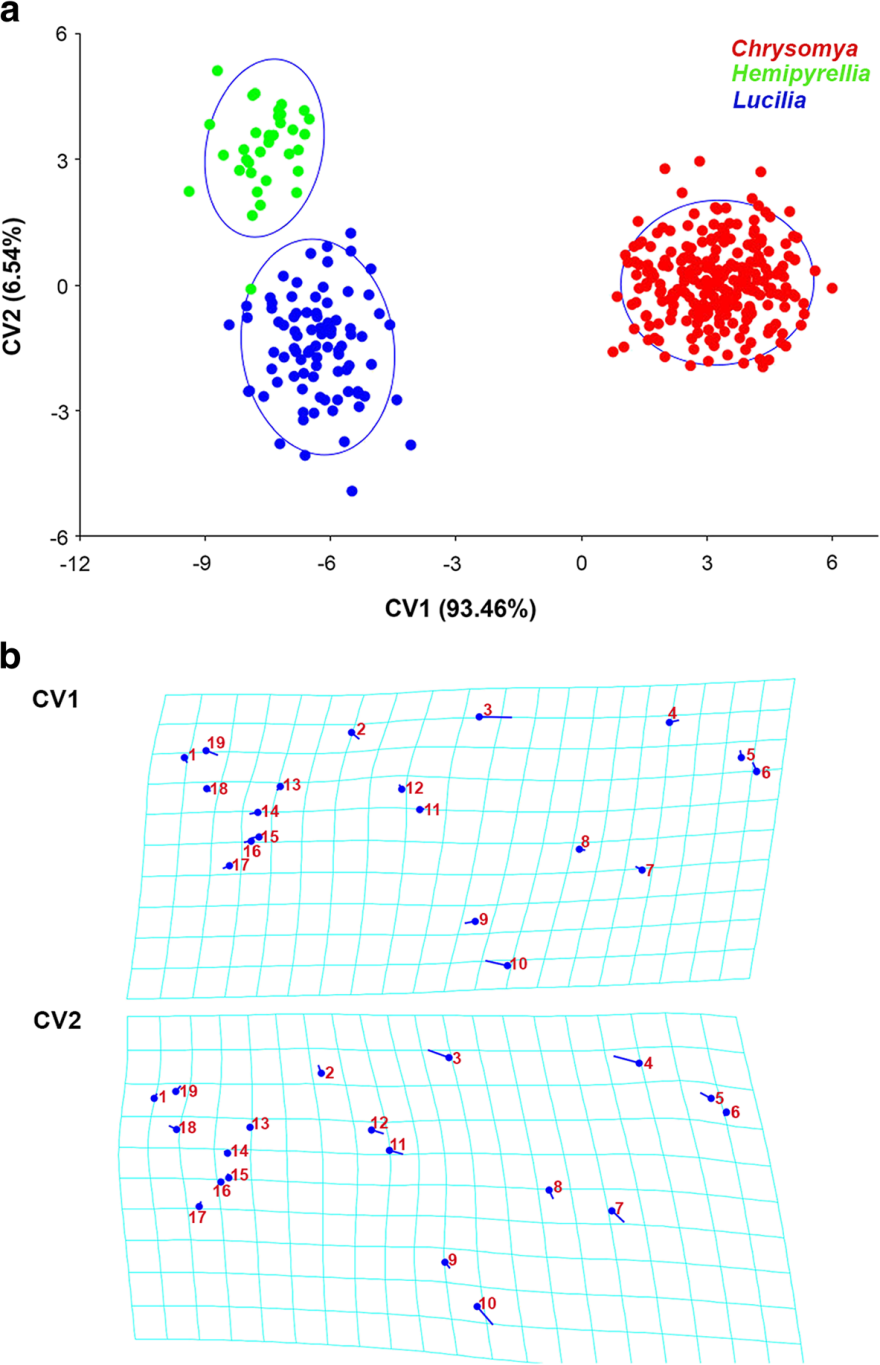
**a** Scatter plot showing the variation in shape of wings of blow fly genera *Chrysomya*, *Lucilia* and *Hemipyrellia* along the first two canonical variate (CV1 and CV2) axes with 90% confidence ellipses. Each genus was clearly separated from the others. **b** Transformation grids illustrate the shape changes from overall mean along CV1 and CV2 axes in positive directions. Circles indicate the locations of the landmarks in the mean shape of the sample; sticks indicate the changes in the relative positions of the landmarks

At the genus level, the first two canonical variates (Fig. 5) accounted together for 100% of the total variation (CV1 = 93.46%, CV2 = 6.54%), and showed that specimens clustered into distinct groups belonging to the same genus, and successfully placed all three genera in their respective subfamily. The scatter plot from CV1 and CV2 (Fig. 5) shows that each genus can be clearly separated from each other. Mahalanobis distances obtained by pairwise comparisons of all three genera revealed highly significant differences (permutation 10,000 rounds in MorphoJ: *P* < 0.0001), ranging from 4.9309 (*Lucilia* and *Hemipyrellia)* to 11.3103 (*Chrysomya* and *Hemipyrellia)* (Table 2). Procrustes distances also showed highly significant differences between genera (permutation 10,000 rounds in MorphoJ: *P* < 0.0001), ranging from 0.0336 (*Lucilia* and *Hemipyrellia*) to 0.0741 (*Chrysomya* and *Hemipyrellia*) (Table 2). Visualised shape changes along CV1 axis were found with landmarks 3, 10, 19, 2, 4, 5, 6 and 9, whereas shape changes along CV2 axis were most clear using landmarks 10, 4, 3, 7, 5, 11 and 12 (Fig. 5). The cross-validation test showed a high percentage of correctly classified specimens with 94.3% (*Hemipyrellia*), 97.7% (*Lucilia*), and 100.0% (*Chrysomya*) (Table 3).

**Table 2:** Difference in wing shapes of blow flies among genera analysed with canonical variate analysis (CVA)

	*Chrysomya*	*Lucilia*	*Hemipyrellia*
*Chrysomya*	–	0.0495	0.0741
*Lucilia*	**9.6888**	–	0.0336
*Hemipyrellia*	**11.3103**	**4.9309**	–

Mahalanobis distances (bold) and Procrustes distances (narrow). *P*-values of all three genera were highly statistically significant (permutation 10,000 rounds in MorphoJ: *P* < 0.0001)

**Table 3:** Percentage of correctly genus-classified specimens by using a permutation test with 10,000 rounds in MorphoJ

Genus	% correctly classified (No. of correctly classified/Total no. of specimens)
*Chrysomya*	100 (250/250)
*Lucilia*	97.7 (85/87)
*Hemipyrellia*	94.3 (33/35)

**Fig. 6:**
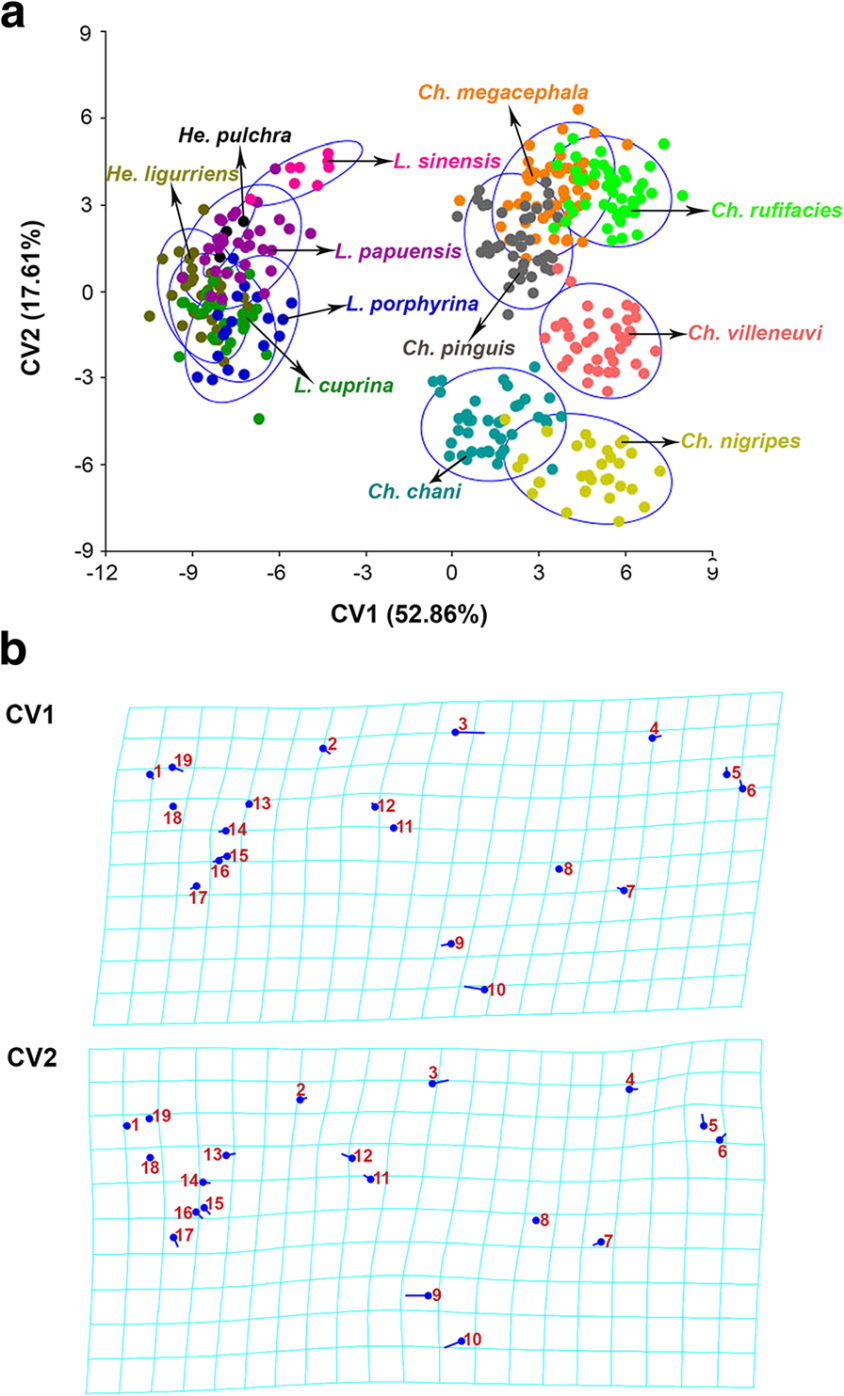
**a** Scatter plot showing the variation in shape of wings of 12 blow fly species along the first two canonical variate (CV1 and CV2) axes with 90% confidence ellipses. **b** Transformation grids illustrate the shape changes from overall average along CV1 and CV2 axes in positive directions. Circles indicate the locations of the landmarks in the mean shape of the sample; sticks indicate the changes in the relative positions of the landmarks

At the species level, the first two canonical variates (Fig. 6) accounted together for 70.47% of the total variation (CV1 = 52.86%, CV2 = 17.61%), showing that specimens were clustered into distinct groups in accordance with their species. The scatter plot from CV1 and CV2 (Fig. 6) showed overlap among species, especially between species in the same subfamily. Species within Luciliinae showed larger overlap than the species within Chrysomyinae. Most of the Mahalanobis distances obtained by pairwise comparisons of the 12 blow fly species were significantly different (permutation 10,000 rounds in MorphoJ: *P* < 0.0001 and *P* < 0.01), ranging from 4.9507 (*Ch. pinguis* and *Ch. megacephala*) to 16.3182 (*Ch. nigripes* and *He. pulchra*) (Table 4). Procrustes distances also showed highly significant differences between most of the species (permutation 10,000 rounds in MorphoJ: *P* < 0.0001, *P* < 0.01, and *P* < 0.05), ranging from 0.0227 (*Ch. pinguis* and *Ch. megacephala*) to 0.0994 (*Ch. rufifacies* and *He. ligurriens*) (Table 4). Visualised shape changes along CV1 axis were found with landmarks 3, 10, 19, 2, 4, 5, 6 and 9, whereas shape changes along CV2 axis were most clear using landmarks 9, 10, 3, 17, 12 and 5 (Fig. 6). The cross-validation test showed a high percentage of correctly classified specimens in most species (> 70%), except for *He. pulchra* (33.3%) (Table 5).

**Table 4:** Difference in wing shapes among 12 blow fly species analysed with canonical variate analysis (CVA)

Species	CM	CC	CP	CN	CR	CV	LC	LPA	LPO	LS	HL	HP
CM	–	0.0493***	0.0227***	0.0404***	0.0237***	0.0442***	0.0730***	0.0561***	0.0681***	0.0366***	0.0871***	0.0755***
CC	9.8564***	–	0.0362***	0.0403***	0.0654***	0.0329***	0.0510***	0.0400***	0.0417***	0.0457***	0.0595***	0.0536**
CP	4.9507***	9.2418***	–	0.0359***	0.0380***	0.0313***	0.0619***	0.0451***	0.0522***	0.0305***	0.0734***	0.0648**
CN	11.4708***	10.7335***	10.4253***	–	0.0503***	0.0441***	0.0660***	0.0560***	0.0613***	0.0469***	0.0808***	0.0747**
CR	5.9621***	10.9845***	7.1383***	11.4291***	–	0.0578***	0.0858***	0.0700***	0.0820***	0.0451***	0.0994***	0.0874***
CV	7.8729***	8.4819***	6.3717***	8.5035***	7.4158***	–	0.0574***	0.0447***	0.0563***	0.0490***	0.0674***	0.0539***
LC	13.5404***	12.4207***	12.7527***	15.8552***	15.1926***	14.7910***	–	0.0259***	0.0339***	0.0520***	0.0319***	0.0347**
LPA	12.1571***	12.7886***	10.8236***	15.3404***	14.0886***	13.7671***	8.3257***	–	0.0284**	0.0340**	0.0397***	0.0369*
LPO	12.8250***	11.5912***	10.8626***	14.3585***	14.7295***	13.4374***	8.5241***	5.6018***	–	0.0444***	0.0344***	0.0451**
LS	11.0842***	13.9298***	10.2695***	15.3045***	12.4569***	13.7332***	10.5660***	5.5405***	8.3450***	–	0.0655***	0.0598**
HL	13.7576***	13.1727***	12.2678***	15.9458***	15.1906***	14.7735***	7.6458***	7.0393***	5.5794***	9.8839***	–	0.0276**
HP	12.6284***	13.3789***	11.9967**	16.3182***	14.4229***	14.6569***	8.2272**	6.2182**	7.5083**	8.2701**	6.0139***	–

Mahalanobis distances (bold) and Procrustes distances (narrow)

*P*-values of all 12 species indicate highly significant differences denoted with asterisks (permutation 10,000 rounds in Morpho J: ***P < 0.0001; **P < 0.01; *P < 0.05).  *Abbreviations*: CC, *Ch. chani*; CM, *Ch. megacephala*; CN, *Ch. nigripes*; CP, *Ch. pinguis*; CR, *Ch. rufifacies*; CV, *Ch. villeneuvi*; LC, *L. cuprina*; LPA, *L. papuensis*; LPO, *L. porphyrina*; LS, *L. sinensis*; HL, *He. ligurriens*; HP, *He. pulchra*

**Table 5:** Percentage of correctly classified specimens in each blow fly species and between sexes of each species performed by using a permutation test with 10,000 rounds in MorphoJ

Species	% correctly classified between species (No. of correctly classified/Total no. of specimens)	% correctly classified between sexes (No. of correctly classified/Total no. of specimens)	
		Males	Females
Ch. megacephala***	98.1 (52/53)	87.5 (21/24)	89.7 (26/29)
Ch. chani***	100 (40/40)	100 (17/17)	100 (23/23)
*Ch. pinguis*	97.4 (38/39)	62.1 (18/29)	50 (5/10)
*Ch. nigripes*	90.6 (29/32)	82.4 (14/17)	73.3 (11/15)
Ch. rufifacies***	97.9 (46/47)	84.0 (21/25)	81.8 (18/22)
Ch. villeneuvi**	100 (39/39)	95.7 (22/23)	100 (16/16)
L. cuprina***	72.4 (21/29)	86.7 (13/15)	78.6 (11/14)
L. papuensis***	71.9 (23/32)	77.8 (7/9)	82.6 (19/23)
L. porphyrina**	83.3 (15/18)	100 (11/11)	71.4 (5/7)
L. sinensis*	75 (6/8)	100 (4/4)	75 (3/4)
He. ligurriens***	87.5 (28/32)	92.9 (13/14)	88.9(16/18)
*He. pulchra*	33.3 (1/3)	–	–

Statistically significant differences between males and females based on Mahalanobis distances are denoted with asterisks (permutation 10,000 rounds in MorphoJ: ***P < 0.0001; **P < 0.01; *P < 0.05). *Hemipyrellia pulchra* has only females, thus it could not be used for classifying between sexes

### Sexual shape dimorphism

The DFA in wing shape between males and females of most species revealed highly significant differences (permutation 10,000 rounds in MorphoJ: *P* < 0.0001, *P* < 0.01, and *P* < 0.05), except for *Ch. pinguis* and *Ch. nigripes* (permutation test with 10,000 rounds in MorphoJ: *P* > 0.05). Moreover, the percentage of correctly classified specimens after a cross-validation test ranged from 50% (*Ch. pinguis*) to 100.0% (*Ch. chani*) (Table 5).

### Phenetic relationships of wing shape among blow fly species

**Fig. 7:**
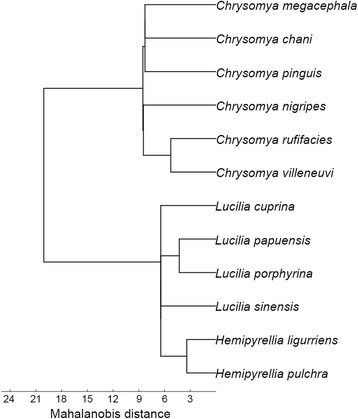
UPGMA dendrogram showing phenetic relationships of wing morphology among blow fly species constructed based on the Mahalanobis distances between species

The UPGMA dendrogram analysis revealed that the 12 blow fly species were divided into two distinct groups, comprising the subfamilies Chrysomyinae (*Chrysomya* spp.) and Luciliinae (*Lucilia* spp., *Hemipyrellia* spp.). Within Chrysomyinae, *Chrysomya* was separated into two subgroups, and the first one includes *Ch. megacephala* + *Ch. chani* + *Ch. pinguis* and the second one *Ch. nigripes* + (*Ch. rufficacies* + *Ch. villeneuvi*) (Fig. 7). Within the Luciliinae four subgroups were separated, including *L. cuprina*, *L. papuensis* + *L. porphyrina*, *L. sinensis*, and *He. ligurriens* + *He. pulchra*.

### Allometric effects

Regression of the Procrustes coordinates on centroid size among species showed a highly significant difference (permutation test with 10,000 rounds in MorphoJ: *P* < 0.0001), allometry explained 2.3% of total shape variation. The relationship between shape and size within each species showed that wing shape variation was significantly correlated to size in most species (permutation test with 10,000 rounds in MorphoJ: *P* < 0.05), except for *Ch. nigripes*, *Ch. villeneuvi*, *L. papuensis*, *L. porphyrina* and *He. pulchra* (permutation test with 10,000 rounds in MorphoJ: *P* > 0.05) (Table 6). Although the regression of shape variation on size was significant, the percentage of variation in wing shape explained by size changes was relatively low. Additionally, allometry causes significant differences between sexes in *Ch. chani*, *Ch. megacephala*, and *Ch. villeneuvi* (Table 6).

**Table 6:** Percentage of predicted indicates the amount of size-related shape variation of wings in each blow fly species and between sexes of each species

Species	% predicted within species	*P*-value	% predicted between sexes	*P*-value
*Ch. megacephala*	14.3	< 0.0001	18.7	< 0.0001
*Ch. chani*	5.9	0.0438	6.2	0.0082
*Ch. pinguis*	8.1	0.0148	5.0	0.0685
*Ch. nigripes*	4.8	0.1345	5.5	0.0741
*Ch. rufifacies*	11.8	0.0021	3.8	0.0663
*Ch. villeneuvi*	4.8	0.0738	6.2	0.0134
*L. cuprina*	11.8	0.0084	4.9	0.1544
*L. papuensis*	1.2	0.7741	3.0	0.4318
*L. porphyrina*	7.5	0.2559	5.1	0.5069
*L. sinensis*	36.5	0.0366	22.1	0.1269
*He. ligurriens*	12.2	0.0057	4.1	0.2228
*He. pulchra*	59.4	0.1612	–	–

Multivariate regressions of the Procrustes coordinates on centroid size of wings within each species and between sexes of each species performed using a permutation test with 10,000 rounds in MorphoJ

After removing the effect of size on shape variation, a cross-validation test showed a high percentage of correctly classified specimens at the generic level with 94.3% (*Hemipyrellia*), 97.7% (*Lucilia*), and 100.0% (*Chrysomya*). Based on the species level, the cross-validation test also showed a high percentage of correctly classified specimens in most species (>75%), except for *L. sinensis* (62.5%) and *He. pulchra* (0%) (Table 7). Additionally, the results of shape variation between sexes after removing the allometric effects, showed wing shape between males and females were not significantly different in most species (permutation 10,000 rounds in MorphoJ: *P* > 0.05), apart from *Ch. chani*, *Ch. megacephala*, *Ch. rufifacies* and *Ch. villeneuvi* (permutation 10,000 rounds in MorphoJ: *P* < 0.0001 and *P* < 0.01). The percentage of correctly classified specimens between males and females of each species after a cross-validation test ranged from 35.3% (*Ch. nigripes*) to 100.0% (*Ch. chani*) (Table 7).

**Table 7:** Percentage of correctly classified specimens in each species and between sexes of each blow fly species performed using a permutation test with 10,000 rounds in MorphoJ

Species	% correctly classified between species (No. of correctly classified/Total no. of specimens)	% correctly classified between sexes (No. of correctly classified/Total no. of specimens)	
		Males	Females
Ch. megacephala***	96.2 (51/53)	91.7 (22/24)	96.6 (28/29)
Ch. chani***	97.5 (39/40)	100 (17/17)	100 (23/23)
*Ch. pinguis*	94.9 (37/39)	65.5 (19/29)	50 (5/10)
*Ch. nigripes*	87.5 (28/32)	35.3 (6/17)	53.3 (8/15)
Ch. rufifacies***	97.9 (46/47)	84 (21/25)	86.4 (19/22)
Ch. villeneuvi**	100 (39/39)	95.7 (22/23)	100 (16/16)
*L. cuprina*	79.3 (23/29)	53.3 (8/15)	64.3 (9/14)
*L. papuensis*	78.1 (25/32)	44.4 (4/9)	69.6 (16/23)
*L. porphyrina*	94.4 (17/18)	54.5 (6/11)	42.9 (3/7)
*L. sinensis*	62.5 (5/8)	75 (3/4)	25 (1/4)
*He. ligurriens*	75 (24/32)	64.3 (9/14)	38.9 (7/18)
*He. pulchra*	0 (0/3)	–	–

Statistically significant differences between males and females based on Mahalanobis distances are denoted with asterisks (permutation 10,000 rounds in MorphoJ: ***P < 0.0001; **P < 0.01). *Hemipyrellia pulchra* has only females, thus it could not be used for classifying between sexes

## Discussion

Wing size is known to be easily affected by environmental factors [27, 34] and our results clearly show that size cannot be used to separate blow fly species. Only *Ch. nigripes* and *L. cuprina* were clearly separated from the other ten species included in the present study by wing size alone. Moreover, the majority of species do not show significant differences between males and females, except for *Ch. rufifacies*, *L. cuprina*, *L. sinensis* and *He. ligurriens,* where males were smaller than females.

In contrast, wing shape showed to be a stable character compared to size [24, 25, 30] and very informative on the phylogenetic and evolutionary relationship of organisms [43–45]. Therefore, it was not surprising to see that our CVA results proved that wing shape could be used to separate medically and forensically relevant blow flies of Thailand, not only at the genus level but also at the species level. However, the latter depends on the genus to which the species belongs. Wing shapes of *Chrysomya* spp. were clearly distinct from *Lucilia* and *Hemipyrellia* species. But while the percentage of correctly classified specimens from the cross-validation test was very high within *Chrysomya* (>90.6%), wing shape largely overlapped within *Lucilia* and *Hemipyrellia* spp., leading to a much lower percentage of correct assignment (33.3–87.5%). It is not surprising that wing shape of most of *Lucilia* species overlaps. The same pattern is seen using morphology; distinguishing among *Lucilia* species is very difficult because most of them look alike [46], and about molecular data, several studies have shown that *Lucilia* species have low interspecific variation among closely related species [20, 47, 48]. In this regard, using a landmark-based characterization of wing morphology is a reliable technique for classifying *Chrysomya* spp., but a much less precise technique to separate *Lucilia* spp. and *Hemipyrellia* spp. The large overlapping among species within Luciliinae shows that wing shape between *Lucilia* spp. and *Hemipyrellia* spp. is very similar to each other. Therefore, using wing morphometric analysis for species identification within Luciliinae should be done carefully and should be performed in combination with additional morphological methods for accurate species identification. Nevertheless, this study demonstrates that a landmark-based analysis of wing morphometry can be a good tool for identification of Thailand blow fly species, as it was already shown in previous studies on South American taxa *Ch. megacephala* and *Ch. albiceps* [33], and *Cochliomyia hominivorax* and *Cochliomyia macellaria* [24].

Our cluster analysis using UPGMA dendrogram based on the wing morphology of the 12 blow fly species clearly placed all species into their respective subfamily, either Chrysomyinae (*Chrysomya* spp.) or Luciliinae (*Lucilia* spp., *Hemipyrellia* spp.). The phenotypic relationships between species of *Chrysomya* detected here are in accordance with their molecular phylogenetic tree [49, 50]. In the molecular analysis, *Ch. rufifacies* and *Ch. villeneuvi* belong to a different clade than other taxa of the genus *Chrysomya*, suggesting the presence of their phylogenetic signal.

As for the Luciliinae, the phenotypic relationships between *Lucilia* spp. and *Hemipyrellia* spp. detected in this study are congruent with molecular studies. *Hemipyrellia* spp. formed a clade within the *Lucilia* spp. that provided strong support for the synonymy of *Hemipyrellia* and *Lucilia* [51]. Such a result suggests that wing morphology could detect some phylogenetic signal in *Lucilia* and *Hemipyrellia.* Thus, a landmark-based morphometric analysis of wings could be used as a valuable tool in taxonomy and systematics. In comparison with molecular techniques, a landmark-based analysis of wing morphology is simple, reliable and inexpensive, and just requires non-damaged wings for analysis.

Our allometric analysis suggests that wing size explained part of the variation in wing shape. However, the percentage of total variation in wing shape explained by changes in size was very low (2.3%). Thus, allometric effects seem not to be the main factors for shape variation among species. We also found intraspecific allometric effects in most analysed taxa, indicating that size-related shape changes varied among individuals within the same species. In addition, there were also significant allometric differences between sexes of *Ch. chani*, *Ch. megacephala* and *Ch. villeneuvi*. Due to its significant impact, it was important to perform the shape analysis with allomery-free variables. When removing the effect of size on shape variation, the percentage of correctly classified specimens among genera remained the same. The percentage of correctly classified specimens among species, however, decreased slightly. These results show that the removal of allometric effects does not improve species separation and that allometry is not an important factor in wing shape variation among species. Moreover, sexual shape dimorphism was often found in most species when allometric effects were included, but less relevant after removing the allometric effects. In general, male wings were narrower when compared to female wings. Similar results have been reported in blow flies, *Co. hominivorax* and *Co. macellaria,* which sexual shape dimorphism showed that male wings were narrower than female wings in both species [24]. The study of Hall et al. [28] showed significant sexual shape dimorphism in *Chrysomya bezziana*. This suggests that allometry is an important factor of sexual shape dimorphism in wings, which is commonly found in other insects [42]. Therefore, the estimation of the allometric effects is a necessary step in any study of phenotypic variation.

Due to a small number of specimens for *L. sinensis* and *He. pulchra* in this study, further studies including more specimens of these two species are recommended to increase the reliability of wing shape for species discrimination. Although wing landmark-based analysis can be a time-consuming process (e.g. in locating the landmarks for a large-scale study) this technique is simple and high reliable. The reliability of wing morphometric analysis depends on (i) wing preparation, i.e. wings should always be processed in the same way, with flattened slide-mounted wings providing the most accurate method of wing measurement [28]; (ii) morphometric analysis, e.g. using the same photographic equipment with the same conditions, operated by the same person to produce the data, the user should have some skills in collecting landmark coordinates, and digitization should be repeated at least once to reduce the measurement error by averaging the repeated digitizations [35, 38].

## Conclusions

Our results demonstrate that a landmark-based analysis of wings could be used to separate medically and forensically relevant blow flies of Thailand at both genus and species levels, even though it is performed with and without the effects of allometry. Using wing landmarks was a highly reliable method for classifying *Chrysomya* species, but less reliable for species discrimination of *Lucilia* and *Hemipyrellia*. Allometry did not affect species separation but had an impact on sexual shape dimorphism. Therefore, an estimation of possible allometric effects is a necessary step in any study of phenotypic variation by morphometrics methods. The use of wing morphometric analysis could be an alternative method used for both species and sex discrimination. In addition, the congruence between wing morphometric analysis in the present study and molecular phylogenetic tree from the previous studies, suggest that wing morphology is a valuable tool in taxonomy and systematics.

## Additional file

Additional file 1: Detail of the semi-automatic funnel trap. (DOCX 14 kb)

## Abbreviations

CV1: Canonical variate axis
CV2: Canonical variate axis
CVA: Canonical variate analysis
DFA: Discriminant function analysis
PMI_min_: Minimum time since death
SSD: Sexual size dimorphism
SShD: Sexual shape dimorphism
UPGMA: Unweighted pair-group method with arithmetic averages
