# Supplementary material for: Wing morphometrics as a tool in species identification of forensically important blow flies of Thailand
Source: Parasit Vectors. 2017 May 10;10:229. doi: 10.1186/s13071-017-2163-z (PMC5424331; doi:10.1186/s13071-017-2163-z)
Supplement: Additional file 1: — Detail of the semi-automatic funnel trap. (DOCX 14 kb) [file 13071_2017_2163_MOESM1_ESM.docx]

**Additional file 1:** Detail of the semi-automatic funnel trap

The trap consists of five basic parts: (1) an external metal case (40×40×60 cm), (2) a black fly net (36×36×85 cm) stretched on the external metal case (3) a square funnel made of transparent plastic board and fitted with an elastic waistband to the black fly net, acting as a fly entrance module, (4) a timer, and (5) a CD player tray. ~300 g of 1-day-old beef offal was put in a plastic container as an attracting bait and placed at the base of the trap. The timer controlled the operation of the semi-automatic funnel trap. When time reached, the entrance module slid down and closed the fly entrance. Therefore, all captured flies were retained inside the fly cage. Afterwards, the fly net was manually removed from the external metal case and its entrance sealed with an elastic band to prevent the flies from escaping. Then, fly net was transported back to the laboratory at Department of Parasitology, Faculty of Medicine, Chiang Mai University within 1 hour and frozen at -20°C for 2 hours. The trap can be re-used by replacing the attractive bait and the fly net.
